# CNNcon: Improved Protein Contact Maps Prediction Using Cascaded Neural Networks

**DOI:** 10.1371/journal.pone.0061533

**Published:** 2013-04-23

**Authors:** Wang Ding, Jiang Xie, Dongbo Dai, Huiran Zhang, Hao Xie, Wu Zhang

**Affiliations:** 1 School of Computer Engineering and Science, Shanghai University, Shanghai, People’s Republic of China; 2 Institute of Systems Biology, Shanghai University, Shanghai, People’s Republic of China; 3 Department of Mathematics, University of California Irvine, Irvine, California, United States of America; 4 College of Stomatology, Wuhan University, Wuhan, People’s Republic of China; Uni. of South Florida, United States of America

## Abstract

**Backgrounds:**

Despite continuing progress in X-ray crystallography and high-field NMR spectroscopy for determination of three-dimensional protein structures, the number of unsolved and newly discovered sequences grows much faster than that of determined structures. Protein modeling methods can possibly bridge this huge sequence-structure gap with the development of computational science. A grand challenging problem is to predict three-dimensional protein structure from its primary structure (residues sequence) alone. However, predicting residue contact maps is a crucial and promising intermediate step towards final three-dimensional structure prediction. Better predictions of local and non-local contacts between residues can transform protein sequence alignment to structure alignment, which can finally improve template based three-dimensional protein structure predictors greatly.

**Methods:**

CNNcon, an improved multiple neural networks based contact map predictor using six sub-networks and one final cascade-network, was developed in this paper. Both the sub-networks and the final cascade-network were trained and tested with their corresponding data sets. While for testing, the target protein was first coded and then input to its corresponding sub-networks for prediction. After that, the intermediate results were input to the cascade-network to finish the final prediction.

**Results:**

The CNNcon can accurately predict 58.86% in average of contacts at a distance cutoff of 8 Å for proteins with lengths ranging from 51 to 450. The comparison results show that the present method performs better than the compared state-of-the-art predictors. Particularly, the prediction accuracy keeps steady with the increase of protein sequence length. It indicates that the CNNcon overcomes the thin density problem, with which other current predictors have trouble. This advantage makes the method valuable to the prediction of long length proteins. As a result, the effective prediction of long length proteins could be possible by the CNNcon.

## Introduction

It is well known that discovering the three-dimensional (3D) structure of a protein can provide important clues to understand of the mechanism of protein functions. Unfortunately, determination of 3D protein structure through experimental methods, such as X-ray crystallography or NMR spectroscopy, are time consuming and not working effectively with all kinds of proteins, especially membrane proteins [1]. Additionally, there are more than 24 million protein sequences in UniPortKB [2] currently, among which only about 84,508 proteins have had their structures solved experimentally [3]. Furthermore, almost 10,000 entries are newly added into Protein Data Bank (PDB) yearly [3]. That means more than 2,400 years are needed to solve the currently existed protein structures through experimental methods, under the situation of current experimental technology and no more newly discovered proteins. In fact, the number of newly discovered sequences grows much faster than the number of structures solved with experimental methods. The computation method is obviously the only way to bridge the huge protein sequence-structure gap.

Although many 3D protein structure predictors (3D-JIGSAW [4], I-TASSER [5], LOMETS [6], MODELLER [7], MODWEB [8], ROBETTA [9], SWISS-MODEL [10] and so on) with different accuracies have been developed in recent years, few predictors can produce desirable resolution structures for applications in medicine, such as drug design. The latest CASP experiment [11] shows that the progress has slowed and even reaches the bottleneck in direct prediction from one-dimensional (sequence) to three-dimensional (structure). With such difficulties, residue contact maps (CM or residue-residue contact) prediction, a matrix representation of protein residue-residue contacts, is the most promising one among recently developed prediction ideas.

CM of a protein is a simplified version of the protein structure and provides a new avenue for predicting 3D protein structure [12]. As these two-dimensional representations capture all the important features of a protein fold, the whole complex and difficult 3D structure prediction task can be divided into two steps. That is solving the one-dimensional to two-dimensional prediction firstly and then the final two-dimensional to three-dimensional prediction. This idea of divide and conquer makes the problem much easier and also help reconstruct final 3D structure from predicted contact maps. Protein CM has some advantages below. First, CM conveys strong information about the 3D protein structure. Second, the binary CM nature can be regarded as a classical problem of a two-state classification which has been thoroughly studied. Third, it has been shown that the empirical reconstruction algorithms are quite insensitive to high levels of random noise in CM, so that it is not necessary to predict all contacts correctly for reconstructing the protein 3D structure [12]–[15]. So far, several contact maps prediction methods, such as NNcon [16], PROFcon [17], SVMcon [18], RECON [19], CMWeb [20] and CMAPpro [21], have been developed successfully.

An improved multiple neural networks based contact map predictor, CNNcon, was proposed in this paper. It’s composed of six input sub-networks and one output network, which forms a two-level cascaded network architecture. All the networks used are standard back-propagation neural networks. For network inputs, different sources of information were mixed and most of them had been used separately in some way before.

## Results and Conclusion

### Assessment of the Prediction Efficiency

To score the efficiency of the CNNcon method, two widely used and accepted statistical indices are introduced. Here, we only sketch these scores that are described in detail in [22]–[25].

The first and most frequently used one is accuracy, also referred to as ‘Specificity’, defined as follows:
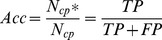
(1)where 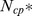 and 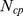 are the number of correctly assigned contacts and that of total predicted contacts respectively. They also correspond to the sum of true positives (TP) and the sum of both TPs and false positives (FT) respectively. Routinely the accuracy is evaluated for each test protein and then averaged over the protein set.

We also evaluate the performance on the coverage of correct predicted contacts, also referred to as ‘Sensitivity’, defined as:
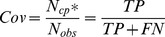
(2)where 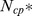 is the same in equation (1) and 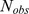 is the number of observed contacts, which corresponds to the sum of TPs and false negatives (FN).

## Results

Table 1 gives the prediction results of sub-networks and the final cascade-network, respectively. Two conclusions follow from these results. First, the prediction accuracy of each sub-network alone is comparable to other neural network based methods [16]–[18], whose performance is showed in Table 2. It indicates that our idea of assigning different prediction tasks to specific sub-networks corresponding to the protein length is practicable. Second, the remarkable improvement of accuracy from final cascade-network with little coverage loss proves that the CNNcon method is extremely effective and valuable.

**Table 1:** Performance of sub-networks and final cascade-network.

	Separation^a^	Seq-Len^b^	THR^c^	Chains^d^	Acc^e^	Err*_acc_* f	Cov^e^	Err*_cov_* f
Sub-network 1	6	51–70	0.1	30	46.43	9.24	41.91	4.89
Sub-network 2	7	71–90	0.6	40	44.35	9.89	36.43	7.64
Sub-network 3	10	91–130	0.7	199	43.99	8.05	36.33	4.59
Sub-network 4	13	131–190	0.7	246	41.38	8.39	33.95	5.32
Sub-network 5	17	191–290	0.8	201	28.31	7.72	36.57	2.64
Sub-network 6	21	291–450	0.9	87	31.81	9.90	34.47	2.53
Average					**34.01**	8.87	**35.44**	4.60
CNNcon		51–450		803	**57.86**	8.07	**34.28**	4.52

a Sequence separation: if value is *s*, then only contacts between pairs 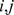 minimally *s* residues apart are considered, that is 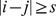.

b Length Range of protein sequence of corresponding sub-network training and testing data sets.

c Minimal prediction value to determine residues contact or not.

d Size of test data set for each sub-network.

e Acc: prediction accuracy(%), defined in equation (1) and Cov: coverage(%), defined in equation (2).

f Standard error.

**Table 2:** Comparison results with other current methods.

Predictor	Acc^e^	Cov^e^	Targets^g^	Method
CNNcon^h^	57.86	34.28	803	Neural network based; Using optimized thresholds.
NNcon^i^	54.50	35.00	116	Neural network based; Top *L*/5 predicted.
PROFcon^j^	32.40	19.60	633	Neural network based; Top *L*/2 predicted.
SVMcon^k^	37.00	21.00	48	Support vector machine based;Top *L*/5 predicted.

e As in Table 1.

g Size of test data sets.

h This work.

i,j,k Results are summarized from previous works [16]–[18], respectively.

In general, it is neither straightforward nor completely fair to compare the performance of different contact map predictors. First, different predictors are usually suitable for different length range proteins. Second, there also not existed a benchmark data set big enough and accepted widely. Therefore, the comparisons with other current contact map predictors in Table 2 are used for reference. The results show that the CNNcon method achieves the best accuracy and the coverage is the second best, which is almost as good as the best one. Moreover, the largest test data set is used in order to make the present results reliable.

To further verify the performance of the CNNcon method, we applied all the compared methods on the same test data set, 64 CASP10 targets. This test data set contains all the targets with length from 51 to 450 and valid PDB codes. Since different methods predict different number of contacts, in order to correctly compare them, *n* predicted contacts with the highest probabilities are selected. To increase the comparison preciseness, instead of being assigned one value, *n* was assigned to *T*/2, 2*T*/3 and *T*, respectively, where *T* was the total true contacts of the whole test data set. Then the final compared statistical indices take the average values. The details of the compared results are given in Table 3. Both accuracy and coverage of the present method are better than others.

**Table 3:** Comparison results on 64 CASP10 targets.

Predictor	Acc^e^	Err*_acc_* ^f^	Cov^e^	Err_cov_^f^
CNNcon	55.48	17.13	36.89	4.79
NNcon	46.39	11.79	31.70	9.49
PROFcon	39.90	7.02	25.55	9.87
SVMcon	38.15	9.02	25.62	10.93

e,f As in Table 1.

**Figure 1:**
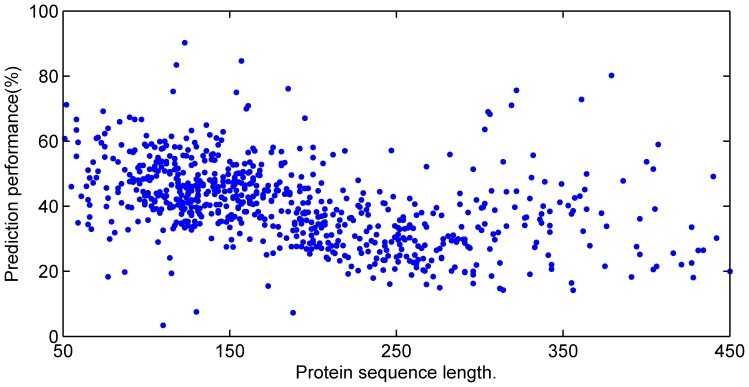
Prediction results upon all test proteins by corresponding sub-networks. The X axis is length range of tested proteins. The Y axis is prediction accuracy (%). Each point represents the predicted accuracy of a protein by its belonged sub-network. The average accuracy is as high as 34.01%. However, the accuracies decrease while the length of proteins increases.

**Figure 2:**
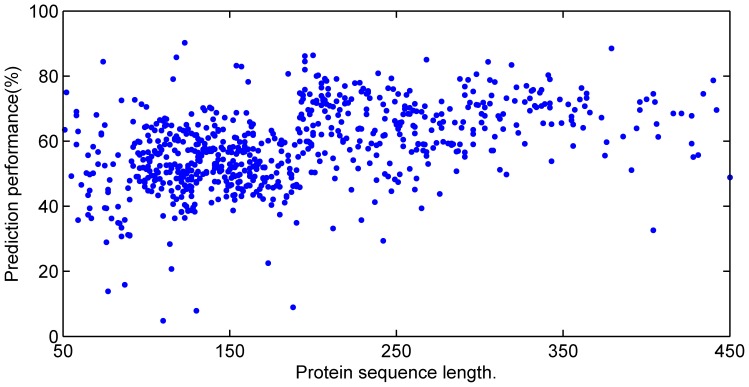
Prediction results upon all test proteins by the final cascade-network. The X axis and Y axis are the same in Figure 1. Each point represents the predicted accuracy of a protein by the final cascade-network. The average accuracy is as high as 57.86%. Moreover, the accuracies keep steady while the length of proteins increases.

The prediction accuracies upon all proteins in the six test sets by corresponding sub-networks are shown in Figure 1. Clearly, accuracies decrease sharply while protein sequence length increases owing to the density of contacts decreasing greatly as the inverse of the protein length [12], [26]. This also troubles most other current contact predictors. However, the prediction accuracies from the present method almost keep the same with the increase of protein sequence length in Figure 2. That means the CNNcon method overcomes the thin density problem [12], [26], which suggests that it might be a valuable candidate for long length protein prediction.

### Conclusion

An improved neural network based approach for protein contact map prediction, called CNNcon, was developed in this paper. The method performs better on prediction accuracy than other compared state-of-the-art methods. Further, the CNNcon method has better consistency and stability on prediction accuracy as protein length increases. Although training the six sub-networks and one cascade network costs computationally more than single-network predictors, it is one-time work. While in testing, the CNNcon method can divide the contact map prediction task naturally and run in parallel, based on the specially designed architecture. This advantage makes the method almost as fast as other single network based methods. It is expected that the CNNcon will be used to enhance parallel performance with longer protein length. As the neural network can be improved by adding more input information and training with a larger training data set, next work will be focus on combining more input information (e.g. correlated mutation information) and adding more protein chains to training data set. Parallel version of the CNNcon algorithm will also be implemented and worked on supercomputers in the future.

## Discussion

### Optimized Thresholds were Crucial for Performance

Table 1 (Column ‘THR’) gives the optimized thresholds for all sub-networks. They are minimal prediction values to determine residues contact or not for corresponding sub-networks. Different thresholds resulting in both different accuracies and different coverages are found. And different sub-networks have their own optimized thresholds. This was probably related to the different contact densities of different protein length ranges, according to which the sub-networks were introduced. Further, it is discovered that the coverage score dropped sharply while the threshold was once greater than a specific value. These specific values were used as our final optimized thresholds for the corresponding sub-networks.

### Combining and Balancing Multiple Predictions Improves Accuracies

As expected, the prediction accuracies of sub-networks are at the same level of most single neural network based methods. However, the final prediction accuracy is improved greatly by our cascade-network because of the following two advantages of our model. First, instead of being processed by a single network, each test protein was input to its corresponding sub-network, left-next sub-network and right-next sub-network for prediction in parallel. This increases the opportunity of contacted amino acids to be found. Second, three optimized balancing weights were introduced to balance the predicted results of sub-networks during final cascade-network prediction.

## Materials and Methods

### Contact Map Definition

The contact map of a protein with *N* amino acids is an 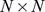 binary symmetric matrix 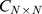. The components 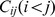 are defined as follows:
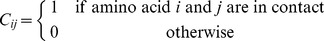
(3)


We define two amino acids as being in contact if the distance between their 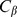 atoms (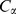 for glycines which having a hydrogen substituent as its side-chain) is less than 8 Å, a standard threshold widely used [12], [22], [27]–[29].

### Neural Network Architecture

**Figure 3:**
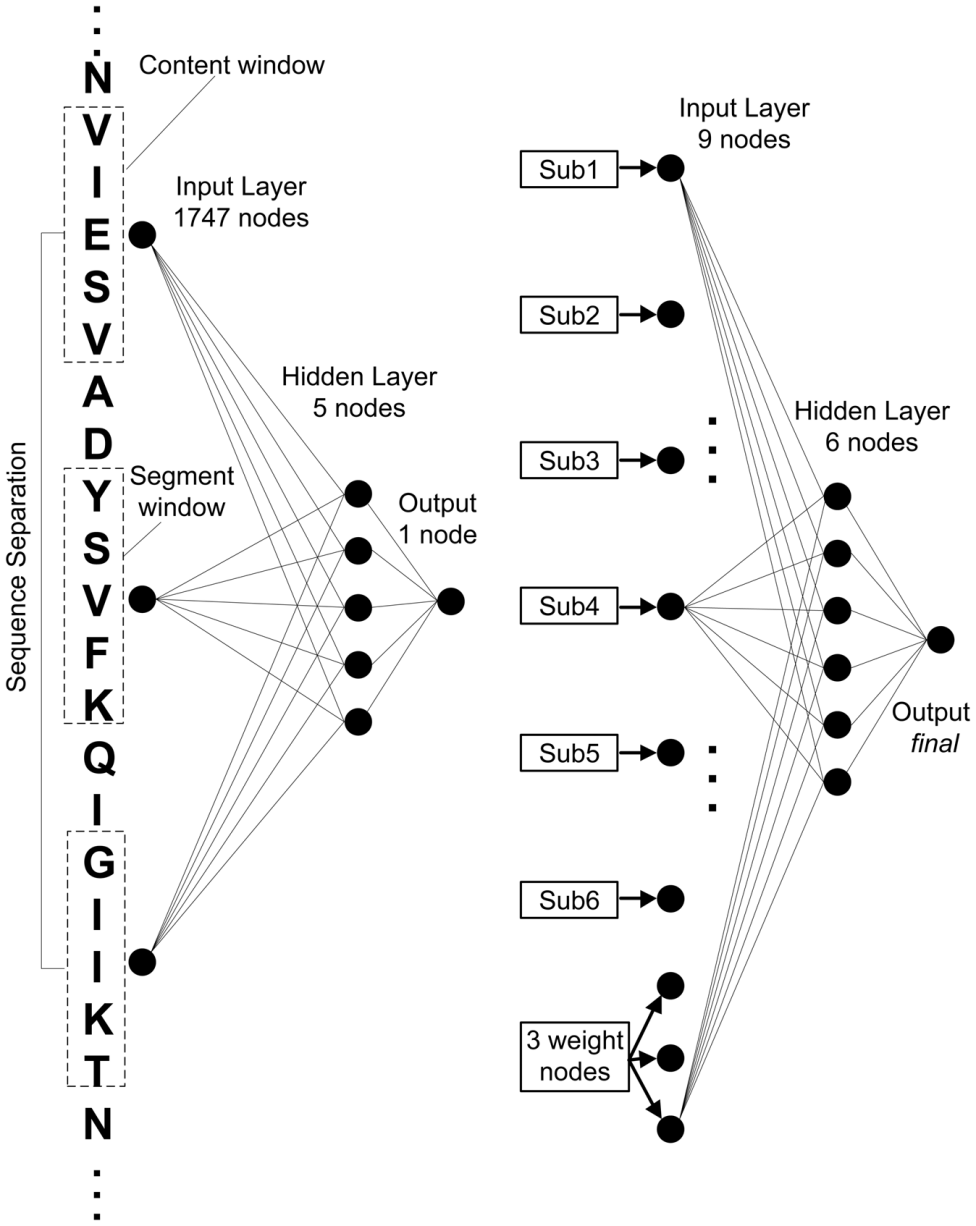
Architectures of sub-neural network (left) and cascade-neural network (right). Since architectures of all the six sub-networks are the same, only one of them is shown here (left).

The finding that number of contacts in a protein is proportional to the protein length *N*, while the number of possible contacts increases with 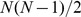 [12], implies the contact densities in the map decrease as the inverse of the protein length. In other words, long proteins have lower contact densities than short ones [26]. This makes the contact maps of long proteins more difficult to predict and the prediction accuracy is affected by the protein length greatly. Six specific sub-networks for different protein length range respectively and one cascade-network are introduced in order to solve this problem. They are all classical feed-forward 3-layer neural networks trained with the same standard back-propagation algorithm [30]. Architectures of all the six sub-networks are the same and composed of 1747 input nodes, 5 hidden nodes and 1 output node. The cascade-network contains 9 input nodes, 6 hidden nodes and 1 output node. The numbers of middle nodes are actually decided by repeated trials in experiment depending on the balance of computation time and prediction accuracy. Same values are assigned to the number of middle nodes of all sub-networks. In fact, it might be more suitable to assign the parameter of each sub-network with its different and specific values, since each sub-network is designed for proteins with different lengths. In next improved version of CNNcon (v2.0, also parallel and super computer version), this work will be considered and these optimal values of this parameter will be picked out through experiments performed on each sub-network. Among these input nodes, six are coded by prediction results from sub-networks and the remaining three are coded by balanced weights. The whole architecture of the CNNcon method is shown in Figure 3.

Each sub-network was trained and tested with its corresponding data set. The data sets and length range divisions are mentioned in section of data sets below. While for testing, the target protein was coded first and input to its corresponding sub-networks for prediction. We defined the sub-network id as 1 to 6 as increase of its length coverage and the particular sub-network with length range covering the target protein length defined as *i*. Thus for each target protein, its corresponding sub-networks were 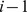, *i* and 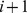, that is just local communication needed while in parallel. After prediction by its corresponding sub-networks, the intermediate results along with three optimized balance factors were input to the cascade-network to finish the final prediction.

### Input Codings

The basic input coding method used here is the same as previously introduced in [31]. Each residue pair is characterized by an vector containing 210 elements 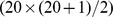, representing all the possible ordered couples of residues. The input coding vectors of each residue couple and its symmetric ones are the same.

In the method, multiple sequence information instead of single sequence was used, since evolutionary information had been proved to improve prediction performance greatly [17]. Multiple sequence alignment information of each protein sequence was gained from its corresponding HSSP file [32]. Considering the prediction performance and our computing resource, we chose as most as 100 multiple sequence alignment sequences (including the target one) with the identity of each aligned sequence less than 80%.

For each sequence in the alignment, a pair of residues in position *i* and *j* were counted. The final input coding, representing the frequency of each pair in the alignment, was normalized to the number of the aligned sequences [31].

Conservation weights and secondary structures [33] information from HSSP file were also coded with one and three elements respectively. Thus the length of the input coding vector becomes 218 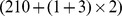.

To obtain local information of each residue, similar to [17], we used two content windows of size 2 centered around *i* and *j* (window of *i*: 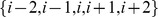, window of *j*: 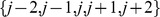) respectively. That means that, for each residue pair 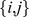, we incorporated information from all residues in those two windows of five consecutive residues. Thus, the length of the input coding vector was increased to 1090 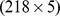.

Further, we introduced a segment window with size of 2 to code information from the segment connecting *i* and *j*. For each residue pair 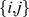, we incorporated information from all residues in the window centered around *k* 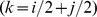, which was the middle position of *i* and *j*. Thus, the segment window spanned the interval 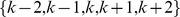) and the length of our input coding vector again was added to 1744 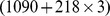.

Finally, we used sequence separation, sequence length and segment separation length to represent the global information from the entire protein. The size of our input coding vector was lastly set to 1747 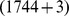.

### Data Sets

Data set used here for training and testing was extracted from the March 2012 25% pdb_select list [34]–[37] with 5,300 chains and 788,447 residues.

For the goal of algorithm design, we removed all protein chains of non-X-ray determined structures, all chains with resolution greater than 1.5 Å, all backbone broken chains (contain missing backbone atoms in the PDB files), all chains containing non-standard residues in its corresponding PDB files and all chains with obsolete PDB ID (e.g. 3G62 is obsolete and replaced by 4F1U). We reduced the data set further by excluding all protein chains longer than 450 residues. Without loss of generality, all chains shorter than 51 residues were removed as well. After above processing, our final data set contains 1,103 chains (1,082 proteins) and 192,640 residues.

As prediction performances greatly depend on protein length distribution, here we give the protein length distribution of data set to make assessment more reasonable. 7.25% of the proteins have a length from 51 to 70 residues (sub-network 1), 8.16% comprise from 71 to 90 residues (sub-network 2); 22.57% from 91 to 130 residues (sub-network 3); 26.84% from 131 to 190 residues (sub-network 4); 22.76% from 191 to 290 residues (sub-network 5); 12.42% from 291 to 450 residues (sub-network 6). These distributions are also the partitions of length range coverage of sub-networks. That’s also why six sub-networks are needed in the CNNcon method. The data set was split into six subsets according to the above length range distributions. Each sub-network was trained with 50 samples randomly selected from its corresponding data set and tested by the remaining. We used all the six test subsets (803 protein chains in total) to test the final cascaded network.

### Balanced Training

To address the extreme disproportion distribution of true (contacts) and false (non-contacts) samples during the training phase, we used balanced training to reduce back-propagation learning cycles [38]. A balancing probability factor was also introduced to further reduce the false samples and the whole training data set size in a random way.
